# Breast carcinoma in a patient with neurofibromatosis type 1 and huge plexiform neurofibroma of the contralateral breast: a case report

**DOI:** 10.1186/s12905-025-03908-8

**Published:** 2025-07-21

**Authors:** Ayla Kouli, Alyaa Alayoubi, Mousa Alali, Maher Saifo

**Affiliations:** 1 Faculty of Medicine, Damascus University, Fayez Mansour Street, Damascus, P. O. Box: 222, Syrian Arab Republic; 2 Department of Oncology, Faculty of Medicine, Albairouni University Hospital, Damascus University, Harasta M5, Damascus, Syrian Arab Republic

**Keywords:** Neurofibromatosis type 1, Breast cancer, Pectus carinatum, Breast enlargement, High risk

## Abstract

**Background:**

Neurofibromatosis type 1 (NF1) is a genetic disorder associated with an increased risk of various cancers, including breast cancer. This report presents a case of a patient with NF1 who had a huge plexiform neurofibroma of the right breast and developed invasive carcinoma in the left breast.

**Case presentation:**

A woman with a known history of NF1, enlarged right breast, and pectus carinatum presented with locally advanced breast cancer of the left breast. The patient underwent four cycles of neoadjuvant chemotherapy with doxorubicin and cyclophosphamide, followed by a left modified radical mastectomy and axillary lymph node dissection. Postoperative pathology showed a complete pathological response. Subsequently, the patient received four cycles of adjuvant paclitaxel followed by endocrine therapy with anastrozole which was replaced by tamoxifen due to bone and muscle pain. At one year follow-up, the patient remains free of disease. The patient was referred to a plastic surgeon for resection of the enlarged right breast.

**Conclusion:**

It is known that women with NF1 have a higher risk of developing breast cancer, as demonstrated in our case. These patients could benefit from an early start of breast cancer screening, which could lead to an early diagnosis of early-stage tumors and a better prognosis. Additionally, enhanced access to healthcare centers and intensive surveillance could contribute significantly to better outcomes.

## Introduction

Neurofibromatosis type 1 (NF1) is a genetic disorder inherited in an autosomal dominant manner, impacting multiple systems and affecting approximately 1 in 3,000 individuals globally. In spite of the full penetrance of *NF1* germline mutations, its clinical presentation is highly variable in terms of severity, rate of progression, and phenotypic expression, making management challenging [1, 2].

The primary manifestations of NF1 include dermatological features such as café-au-lait macules, axillary and inguinal freckling, and the presence of neurofibromas [3]. Studies have shown that patients with NF1 are predisposed to several types of malignant neoplasms, including breast cancer [4–6].

Recent findings suggest that women with NF1 under the age of 50 have up to a fivefold increased risk of developing breast cancer compared to the general population. However, after age 50, the risk in women with NF1 does not differ significantly compared to women without NF1 [4, 7, 8]. Importantly, NF1 is associated with relatively poor breast cancer survival [4, 6]. In this report, we present a case of severe breast enlargement due to a plexiform neurofibroma in association with breast cancer in the contralateral breast.

## Case presentation

A 61-year-old postmenopausal, unmarried woman with a known history of NF1 presented to Albairouni University Hospital in August 2023 with a palpable breast mass. The family history of the patient includes hypothyroidism in both her brother and sister. Additionally, her brother died at age of 29 from lymphoma. Her past medical history includes NF1 diagnosed since childhood and cataract surgery performed two years ago.

**Fig. 1:**
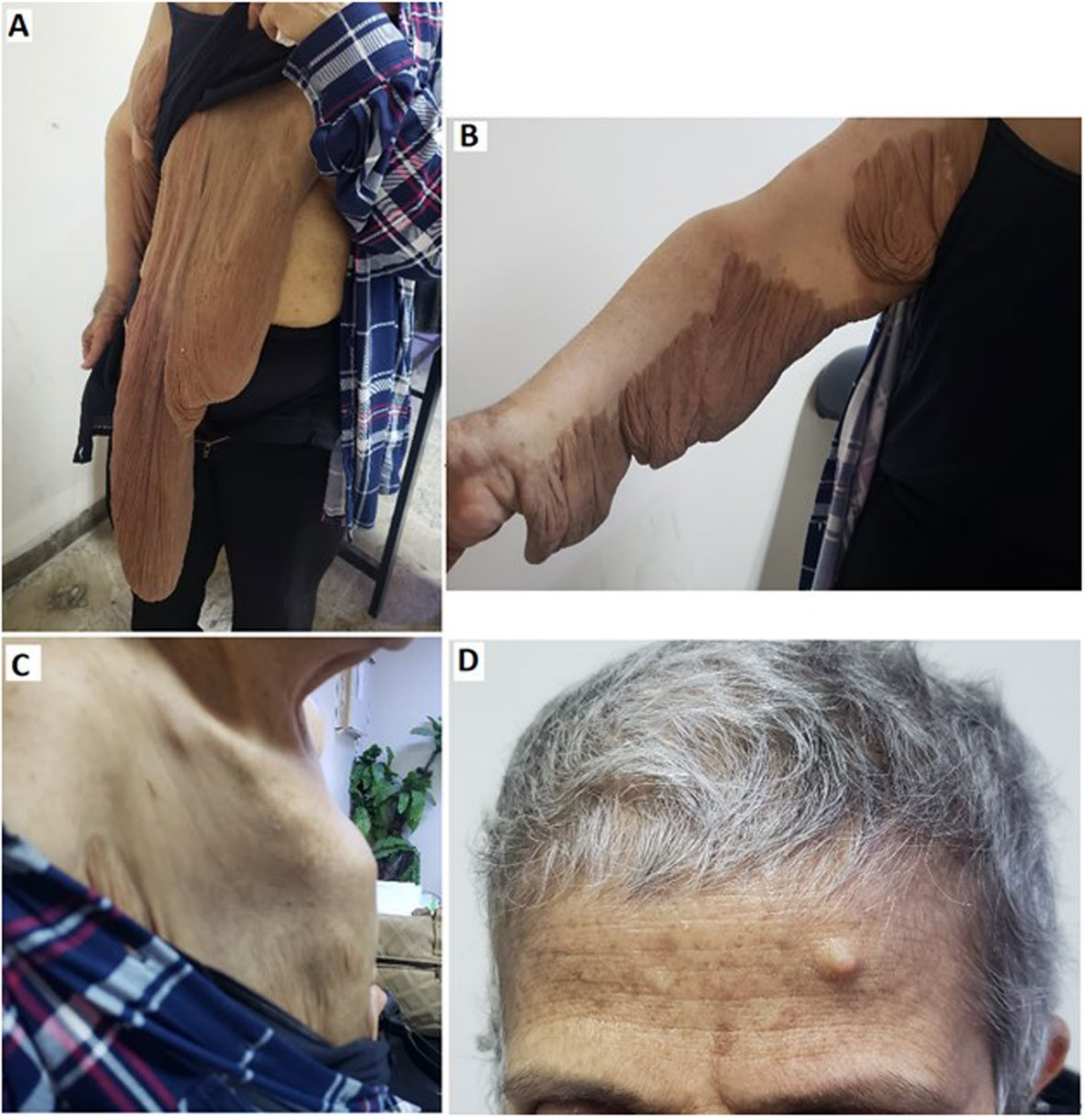
Skin manifestations of a patient with neurofibromatosis type I. **A** Giant plexiform neurofibroma of the breast. **B** Multiple plexiform neurofibromas of the upper extremity. **C** Pectus carinatum, lateral view. **D** Café-au-lait macules with neurofibroma on the forehead

During a surgical consultation for mastectomy of the enlarged right breast, the surgeon incidentally noticed a mass in the left breast. On clinical examination, the patient exhibited pectus carinatum, a gigantic neurofibroma in her right breast, multiple neurofibromas of the upper right extremity, and more than six café-au-lait macules were found on the chest wall and back with numerous freckles distributed over the trunk and axillae (Fig. 1). A mammogram and ultrasound of the left breast revealed an irregular high-density mass measuring 7.6 × 6.4 cm located in the upper outer quadrant; along with an axillary enlarged lymph node measuring 2 × 4 cm. A tru-cut biopsy performed, and pathology revealed invasive lobular carcinoma. A computed tomography (CT) scan revealed increased anteroposterior diameter of the chest consistent with clinically evident pectus carinatum. In addition, nodular thickening of the skin of the right breast extending to the abdominal wall. A hypodense mass with irregular margins measuring 3 × 4.8 cm, accompanied by overlying skin thickening was seen in the left breast. According to the TNM staging, the tumor was classified as cT3N1M0. Immunohistochemical analysis demonstrated that estrogen and progesterone receptor were both positive (4/8), while HER2/neu was negative.

The patient received four cycles of neoadjuvant chemotherapy with doxorubicin and cyclophosphamide. Subsequently, the patient underwent left modified radical mastectomy with left axillary lymph node dissection. Postoperative pathology showed no residual tumor, with free skin, nipple, and surgical margins; in addition to six reactive lymph nodes (Sataloff category Tumor-A and Node-A for the axilla). The patient received four cycles of adjuvant chemotherapy with paclitaxel. After that, endocrine therapy with anastrozole was initiated; however, due to severe muscle pain and osteoporosis, it was discontinued and replaced with tamoxifen at a dose of 20 mg daily.

On a CT scan done one year later, a thyroid nodule was detected with no other suspicious findings. Fine needle aspiration revealed no malignancy. The patient remains free of disease and doing well. The patient was referred to a plastic surgeon for resection of the enlarged right breast.

## Discussion and conclusions

A severe case of NF1 is reported in this report. Despite the well-established association between NF1 and breast cancer, an early detection program was not followed, resulting in an advanced-stage diagnosis. Nonetheless, surgery and medical treatment were administered properly, and the patient did not have any recurrence to date.

In this case, due to the large size of the tumor, chemotherapy was administered prior to surgery resulting in a complete response. Systemic chemotherapy administered before or after surgery does not significantly differ from each other in long-term outcomes according to randomized clinical trials [9, 10]. Systemic therapy has traditionally been beneficial for improving surgical outcomes before surgery. In addition, preoperative systemic therapy may provide valuable prognostic information based on response. While neoadjuvant therapy is most commonly indicated for HER2-positive and triple-negative breast cancer subtypes, it may also be considered in select hormone receptor–positive cases, particularly when tumor burden is high or breast-conserving surgery is desired. A pathologic complete response (pCR) to neoadjuvant therapy can lead to a favorable disease-free and overall survival rate.

Various types of breast malignancies have been reported in the literature, in association with Von Recklinghausen's disease (NF1); with most available studies on this association were case reports [11–16]. A common feature shared between our case and those previously reported is the late presentation to medical care, often due to patients mistakenly identifying the breast tumor as benign manifestations of NF1, such as neurofibromas. The majority of reported cases involve postmenopausal women between the ages of 50 and 75. A summary of these cases is presented in Table 1. A study by Evans et al. of 142 patients with NF-1 and breast cancer showed a higher incidence of contralateral breast cancer and a shorter survival [17].

**Table 1:** Studies that reported synchronous neurofibromatosis type 1 and breast cancer

Study, year	Age (year)	Study Design	Stage	Histology	Molecular Subtype	Treatment	Outcomes
This case	61	Case report	cT3N1M0	ILC	HR-positive/HER2-negative	Neoadjuvant 4 AC, MRM/ALND, then adjuvant paclitaxel followed by endocrine therapy	Complete response Disease free after 1 year
Nakamura M, 1998 [13] *	49	Case report	T2NxMx	Scirrhous carcinoma	NS	Mastectomy followed by chemoendocrine therapy	Lungs, liver, and bone metastasis, as well as a contralateral breast tumor, developed and she died 4 months after surgery
Wilson CH, 2004 [16]	18	Case report	T0N0M0	Male, bilateral ductal carcinoma in situ	NS	Bilateral mastectomies	NS
Posada JG, 2005 [11]	74	Case report	T2N0M0	ILC	HR-positive/HER2-unknown	Modified radical mastectomy and was then treated with tamoxifen for 3 years	No recurrence No significant complication
Natsiopoulos I, 2007 [12]	60	Case report	T3NxM0	Metaplastic carcinosarcoma	HR-negative/HER2-unknown	Surgery then chemotherapy	Disease free after 2.5 years
Alamsamimi M, 2009 [14]	51	Case report	Left breast: T2N0M0 Right breast: T2N0M0	IDC	HR-positive/HER2- positive	Mastectomy, followed by postoperative chemoendocrine therapy	Disease free after 23 months
Salemis NS, 2010 [15]	59	Case report	T2N1a	IDC	HR-positive/HER2- positive	Mastectomy, adjuvant chemotherapy, trastuzumab, and tamoxifen	Disease free after 20 months

*AC* Doxorubicin + cyclophosphamide, *IDC* Invasive ductal carcinoma, *ILC* Invasive lobular carcinoma, *HER2* Human epidermal growth factor receptor 2, *HR* Hormone receptor, *MRM/ALND* Modified radical mastectomy/axillary lymph node dissection, *NS* Not specified

^*^Based on abstract

Mutations within the *NF1* gene on chromosome 17q11.2 result in NF1. The *NF1* gene encodes a tumor suppressor protein called neurofibromin. The *NF1* gene is autosomal-dominant and exhibits complete penetration but a variable expression pattern. 30 to 50% of people with NF1 have unaffected relatives and acquire the germline mutation during embryogenesis de novo [18]. Genetic testing was not performed on the patient in this case because it is not available in public hospitals or covered by insurance. However, genetic testing is not necessary to diagnose NF1.

There is a notable discrepancy in tumor size as measured by mammography/ultrasound versus CT imaging in the current study. This variation is likely due to differences in imaging modality resolution, measurement planes, and tissue contrast characteristics. Ultrasound and mammography, which are breast-specific modalities, are more reliable for primary tumor sizing. It should be noted that, due to resource constraints, no pre-treatment fine-needle aspiration was performed for the enlarged axillary lymph node; therefore, the preoperative axillary lymph nodal status cannot be definitively confirmed as N1. Additionally, some pathological tests were not performed in the current case, including Ki-67 testing, so the tumor could not be classified according to its molecular subtypes (luminal A or luminal B). In this case, initial preoperative treatment consisted of four cycles of chemotherapy. Despite achieving a complete pathological response, adjuvant chemotherapy with four additional cycles of paclitaxel was administered. However, current guidelines suggest that completing the full course of standard neoadjuvant chemotherapy prior to surgery is preferable [19].

In conclusion, this case report illustrates the complex interplay between NF1 (Von Recklinghausen's disease) and breast cancer. The inclusion of NF1 patients in national high-risk breast cancer screening programs is warranted and may substantially improve early detection and survival outcomes in these individuals. Furthermore, improving access to specialized healthcare services and enhancing surveillance in this high-risk population could positively affect prognosis and long-term outcomes.

## Data Availability

The data supporting the findings of this study are available from the corresponding author upon reasonable request.

## Abbreviations

CT: Computed tomography
ILC: Invasive lobular carcinoma
NF1: Neurofibromatosis type 1
